# Mouse model of plasma cell mastitis

**DOI:** 10.1186/1479-5876-10-S1-S11

**Published:** 2012-09-19

**Authors:** Jian-jun Yu, Shan-lin Bao, Sheng-lin Yu, Da-Qing Zhang, Wings TY Loo, Louis WC Chow, Li Su, Zhen Cui, Kai Chen, Li-Qiong Ma, Ning Zhang, Hui Yu, Yun-Zhen Yang, Yu Dong, Adrian YS Yip, Elizabeth LY Ng

**Affiliations:** 1 Department of Surgical-oncology, Affiliated Tumor Hospital, Ningxia Medical University, Ningxia, PRC; 2 Department of Breast and Mini-invasive Surgery, Ningxia People's Hospital, Ningxia, PRC; 3 UNIMED Medical Institute and Organisation for Oncology and Translational Research, Hong Kong SAR; 4 Department of Clinical Oncology, Ningxia Peoples’ Hospital, Ningxia, PRC; 5 Department of Surgical-oncology, the Affiliated Hospital, Bengbu Medical College, Bengbu, PRC; 6 Department of Pathology, the Affiliated Hospital, Ningxia Medical University, Ningxia, PRC; 7 Medical College of Xi’an Jiaotong University, Xi’an, PRC

## Abstract

**Background:**

Plasma cell mastitis is distinct from the common form of mastitis and clinically resembles breast carcinoma. The lesion occurs in non-lactating young women, and the incidence rate is rising. Surgical resection is the main treatment, but cannot prevent recurrence of the disease. Disfigurement or removal of breast after the operations can cause marked physical and psychological distress. The etiology of plasma cell mastitis is unclear up till now. It is therefore necessary to investigate further the underlying immunological changes of the disease.

**Methods:**

The lesions of plasma cell mastitis removed from patients through aseptic operation were mixed with normal saline into homogenate tube machine (homogenate tubes were disinfected and sterilized prior to treatment). The mixture was homogenized at medium speed and grinded in ultrasonic cell disruptor. The homogenate obtained was made into oil emulsion with Freund's adjuvant. Thirty female BALB/c mice (6 weeks after sexual maturity) were divided into five groups A-E: group A was blank control; group B was normal saline control; group C was inoculated with 0.02 ml water-in-oil emulsion; group D was inoculated with 0.04 ml water-in-oil emulsion; group E was complete Freund's adjuvant control.

**Results:**

Pathology results showed that mouse mammary gland acinar cells remained integral without any abnormal changes observed in control groups A and B. Experimental groups C and D showed dilation of mouse mammary ductal tissue with a large number of epithelial cells and debris in the lumen, and fibrosis around ducts accompanied by large duct cells, neutrophils, lymphocytes, and especially plasma cell infiltration. Pathological changes were observed in 3 (50%) mice and 5 (83.3%) mice in group C and D respectively. In group E, neutrophil infiltration in mammary gland was observed in 5 mice, but neither infiltration of plasma cells nor other abnormal pathological changes were observed.

**Conclusions:**

The lesions of patient with plasma cell mastitis could make the female BALB/c mice experience the similar clinical and pathological manifestation. High-dose group can successfully establish a mouse model of plasma cell mastitis.

## Background

Plasma cell mastitis (PCM), also known as granulomatous mastitis or nonpuerperal mastitis, was first proposed by Cheatle and Cutler, in 1931[1]. It was a nonbacterial inflammatory breast disease frequently occurred among young and middle-aged women at non-pregnancy or non-lactation stages [2,3]. According to the current report, the incidence rate of PCM increased gradually, and extended to pubertal or menopausal women [4-7]. However, little is known about its mechanism as its onset is occult, but several studies have shown that recurrence of PCM was commonly observed [8-10]. Surgical excision is the major treatment of the disease [2,10,11] and wide local excision was commonly performed as limited surgical excision might lead to higher rate of recurrence [10]. However, the operation could not prevent the recurrence of the disease as a discrete mass [12,13] even though the mammary gland was removed, thus women might experience mental and physical distress.

The inflammatory disease clinically mimics breast cancer [14,15] and some cases were misguided as breast cancer [16,17]. Histologically, the disease is presented with duct widening characterized by periductal inflammation and fibrosis. Some characteristic calcification can be seen by mammography. Microscopically, granuloma formation, Langhan’s giant cell, plasma cell, histiocyte, lymphocyte, and caseous necrosis were observed [18,19]. However, a prospective follow-up study of 270 patients with nonpuerperal breast inflammation has demonstrated an increased risk of breast cancer in women with nonpuerperal mastitis [20]. The correlation between the inflammatory disease and breast cancer has not yet been established.

In this study, a PCM mouse model was established by inoculation of aseptic homogenate of human PCM pathological tissues and complete Freund’s adjuvant (CFA) to investigate the immunologic mechanism of PCM.

## Methods

### Agents and equipments

Agents and equipments applied in this study included complete Freund’s adjuvant (Sigma-Aldrich Co., Ltd., U.S.A), low temperature refrigerator (Qingdao Haier Co., Ltd, China), electronic balance, ultrasonic cell disruptor, tissue homogenizer and histotome.

### Homogenate

Fresh and noninfected PCM pathological tissues were excised under aseptic conditions. Pathological tissues (0.1g) mixed with physiological saline at a ratio of 1:3 was added to the sterilized homogenate pipe. The samples were homogenated for 5 minutes at intermediate speed. Ice cubes were placed into the shattering slot of the machine to guarantee that the shattering samples were processed at 3 to 4°C. The product was treated with ultrasonic cell disruptor (power: 120 W, intermittent time: 10 seconds, working time: 3 seconds, breaking the number of: 60, total duration: 5 minutes). Then the samples were stored in specimen bottles. For the preparation of water-in-oil emulsion, samples and CFA were mixed with equal volume at 0-4°C followed by repeated mixing with syringe for 20 minutes. The water-in-oil emulsion was stored for further experiments.

### Animals

Thirty sexually mature female BALB/c mice aged 6 weeks were randomly divided into 5 groups: group A (n = 6, control ), group B (n = 6, 0.02 ml physiological saline was injected via the 3rd and 4th pairs of lacteral glands), group C (n = 6, 0.02 ml water-in-oil emulsion was injected via the 3rd and 4th pairs of lacteral glands), group D (n = 6, 0.04 ml water-in-oil emulsion was injected via the 3rd and 4th pairs of lacteral glands) and group E (n = 6, 0.02 ml CFA was injected via the 3rd and 4th pairs of lacteral glands). Group A, B and E were regarded as blank control group, negative control group and positive control group respectively. Group C and D were regarded as experimental group. Animals were sacrificed 1 week after inoculation. Pathological changes of PCM were detected by hematoxylin & eosin staining. Positive results were designated in animals with PCM pathological changes, otherwise negative.

### Embedding

Mice purchased from Beijing Vitalriver Co., Ltd. were housed in the animal breeding room for 1 week. For the anesthesia, 0.3% chloral hydrate solution was injected via intraperitoneal injection. Ethanol (75%) was used for the skin disinfection. Abdominal body hairs of anesthetized mice were removed at the 3rd and 4th pairs of lacteral glands. For the embedding, water-in-oil emulsion, sodium chloride physiological solution and CFA solution were administrated via subcutaneous injection at the 3rd and 4th pairs of lacteral glands of group C, D and E mice respectively.

### Mammary tissue collection

Animals were sacrificed 1 week after embedding to collect the tissues from the 3rd and 4th pairs of lacteral glands. After physiological saline rinsing, the tissues went through formalin (10%) fixation for 24 hours to prepare for the paraffin embedding tissues. Then deparaffinage and washing was performed. The sections were stained with routine hematoxylin & eosin staining.

### Assessments and statistics

The diagnosis of PCM was mainly based on the pathologic diagnosis of specific tissues including plasmocytes clustered in the peripheral tissues of mammary duct and lobules of mammary gland combined with infiltration of neutrophils and lymphocytes. Under the microscope of high power fields (HPF), negative and positive pathological changes of PCM were defined as less than 50 inflammatory cells per HPF and 50 or more inflammatory cells per HPF respectively. Parameters were compared using SPSS 18.0 software. Fisher’s exact test or chi-square test was used to compare the number of positive pathological findings of PCM between groups. *P* < 0.05 was considered as statistically significant.

## Results

### Histological comparison between experimental group and control group

**Figure 1:**
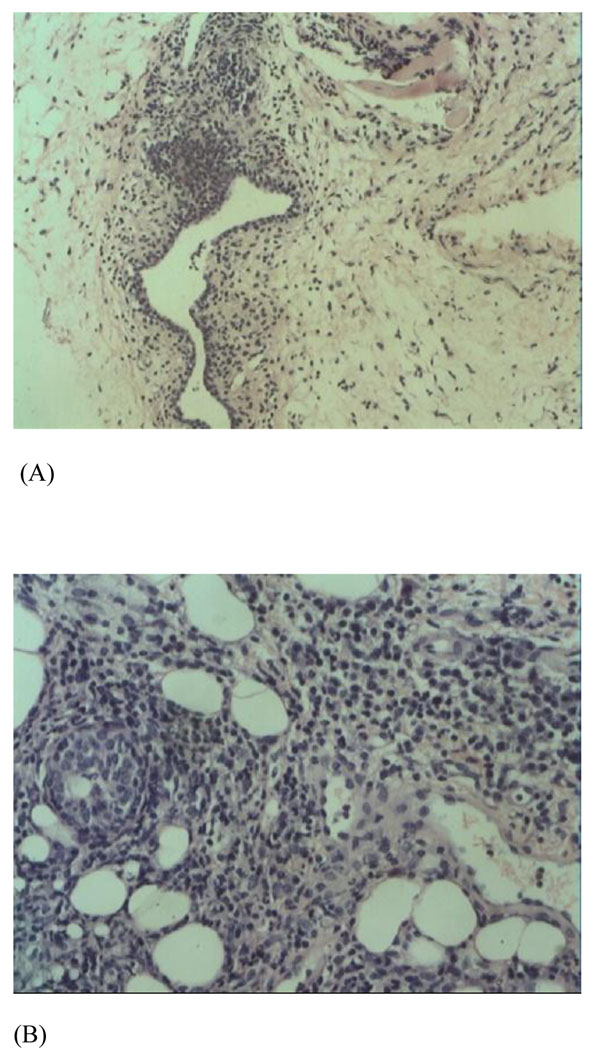
**Histopathological presentation in mouse breast tissues** (A) Control group: No significant plasmocyte infiltration was observed. (B) Experimental group: Infiltration of plasmocytes, neutrophils and lymphocytes was detected.

**Figure 2:**
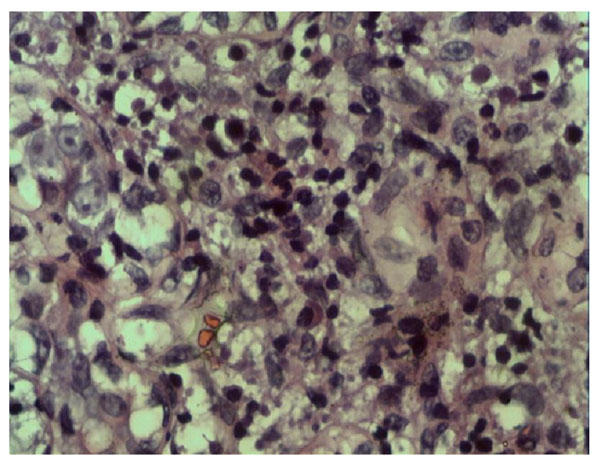
Histopathological presentation in human plasma cell mastitis

Infiltration of plasmocytes, neutrophils and lymphocytes were detected in the peripheral tissues of lobules of mammary gland and mammary duct in the experimental groups C and D, but not in control groups A, B and E (Figure 1). It was similar to histopathological presentation in human plasma cell mastitis (Figure 2). Necrosis was detected in tissues with severe inflammation. No infiltration of plasmocytes, neutrophils and lymphocytes was detected in the control groups. In CFA group, only slight infiltration of neutrophils and lymphocytes were observed, but infiltration of plasmocytes was not observed.

### Pathological comparison between experimental group and control group

In experimental group C and D, swelling and nodules were detected in lacteal gland 1 day and 3 days after injection of water-in-oil emulsion respectively. Pathological samples were taken 1 week after the injection, and abscessation was detected in the lesion regions. Swelling and nodules were not observed in Group A and B. In Group E, a small portion of skin bump, where CFA was not absorbed, existed 1 day after CFA injection but no significant rush node swelling was observed. The bump still existed 3 days later without significant changes. No abscessation was noted at the lesion regions 1 week after the injection. In our study, control groups A, B and E did not show any pathological change of PCM as indicated from sections of tissue with less than 50 inflammatory cells per HPF by microscopic examination. In experimental groups, more than 50% of the mice presented with pathological change of PCM. Significant difference in PCM pathological changes was detected between all groups (P = 0.001) (Table 1). A higher percentage of PCM pathological change was observed in experimental group than control group with a statistical significance (P < 0.0001). Within the experimental group, no difference was detected between group C and group D (P > 0.05) (Table 2), albeit more mice presented with positive pathologic changes of PCM in group D than group C.

**Table 1:** Comparison of positive pathological changes of plasma cell mastitis in different groups

Group	Results		P
		
	Positive	Negative	
A	0 (0.0%)	6 (100.0%)	0.001*
B	0 (0.0%)	6 (100.0%)	
C	3 (50.0%)	3 (50.0%)	
D	5 (83.3%)	1 (16.7%)	
E	0 (0.0%)	6 (100.0%)	

Group A: blank control; Group B: normal saline control; Group C: inoculated with 0.02 ml homogenate; Group D: inoculated with 0.04 ml homogenate; Group E: complete Freund’s adjuvant control.

*Statistically significant by Pearson Chi-Square test

**Table 2:** Comparison of positive pathological changes of plasma cell mastitis in Group C and Group D

Group	Results		P
		
	Positive	Negative	
C	3 (50.0%)	3 (50.0%)	0.545
D	5 (83.3%)	1 (16.7%)	

Group C: inoculated with 0.02 ml homogenate; Group D: inoculated with 0.04 ml homogenate. Fisher’s exact test did not show statistical significance between groups.

## Discussion

PCM is a rare breast disease that mimics breast cancer and the etiology remains unclear. It is therefore clinically significant to differentiate the inflammatory disease from malignant disease. Basically, the disease could be categorized into infectious and non-infectious ones which respectively presented as tuberculous mastitis and idiopathic granulomatous mastitis with distinguishable histological features [4]. However, the immunological mechanism of PCM and its associated pathological change were still unknown. A variety of different terms including granulomatous mastitis, mammary duct ectasia or nonlactational mastitis created unconformity of the designation systems. Previous studies showed anaerobic infection was the major cause of PCM, which might result in tumorigenesis in the infected area [21,22], but the casual relationship between mastitis and breast cancer was not fully supported and widely accepted [23]. Currently, PCM was reported to be associated with nonbacterial infection [24]. Our data indicated that most of the PCM patients showed a history of inverted nipples, which might lead to stenosis and obstruction of mammary duct, as well as deposition of secretions such as breast milk in the mammary duct. We speculated that long-term deposition of breast milk in mammary duct could stimulate autoimmune response, followed by pathological changes in different parts of the mammary gland [3].

Surgery was still the preferred treatment of PCM to the antibiotic treatment which was not efficient. However, complete remission was not guaranteed after surgery. To date, bacterial mastitis mouse models were mainly induced by injection of bacterial liquid via lacteal gland [25] and in a retrospective review of 26 clinical cases of granulomatous mastitis, idiopathic cases were not associated with specific micro-organism with negative special stains and cultures for micro-organisms [26]. In this study, a PCM mouse model was induced by active ingredient of the PCM tissues. The possibility of bacteria induced PCM was eliminated by sterile processing from sample collection to inoculation and ultrasonic cell disruptor processing after homogenization. Moreover, CFA was used to promote the formation of PCM due to the fact that CFA could function as antigen reservoir and stimulate local inflammation and granulomatous reaction as well as the proliferation and differentiation of lymphocytes [27]. The use of CFA in this study could preclude the possibility of positive pathological changes of PCM resulted from enhancement of immunological responses to antigens.

PCM mouse models were established in group C and group D. Infiltration of plasmocytes, neutrophils and lymphocytes was detected in peripheral breast tissues, which was consistent with the diagnostic standard of PCM. No statistical difference of PCM pathological changes was observed between groups. Also, manifestation of PCM was not observed in group A and B, demonstrating that physiological saline did not have any effect on the development of PCM. Infiltration of neutrophils and lymphocytes was detected in group E, indicating that CFA was not efficient in the induction of PCM even though it functioned in promoting inflammation. We therefore confirmed that certain ingredients of pathological tissues from PCM patients could induce the development of PCM in mouse models. The first PCM mouse model presented in this study will provide a platform for the investigation of the etiology, pathological mechanism and the potential drug selection for the PCM.

## Conclusions

The PCM lesion from patients could induce the development of PCM in female BALB/c mice with similar clinical and pathological manifestation and higher dose of PCM homogenate succesfully developed PCM in all mice. The mouse models could be futher investigated to explore the etiology, disease mechanism as well as potential drug therapy while at the moment traumatic surgery is still the major treatment for patients with PCM.

## Competing interests

The author(s) declare that they have no competing interests.

## Authors' contributions

DZ, LS, ZC, KC, LM, NZ, HY, YY, YD, and JY, SB, and SY carried out the scientific research. JY, WTYL, LWCC, AYSY and ELYN participated in the writing of the manuscript. WTYL and AYSY performed statistical analysis. JY and LWCC provided expert opinion for the study. JY, SB, and SY were involved in consultation for the research. JY was the initiator of the study.
